# Supplementary figures and images for: Mechanically stimulated osteocytes maintain tumor dormancy in bone metastasis of non-small cell lung cancer by releasing small extracellular vesicles
Source: eLife. 2024 Mar 28;12:RP89613. doi: 10.7554/eLife.89613 (PMC10977966; doi:10.7554/eLife.89613)

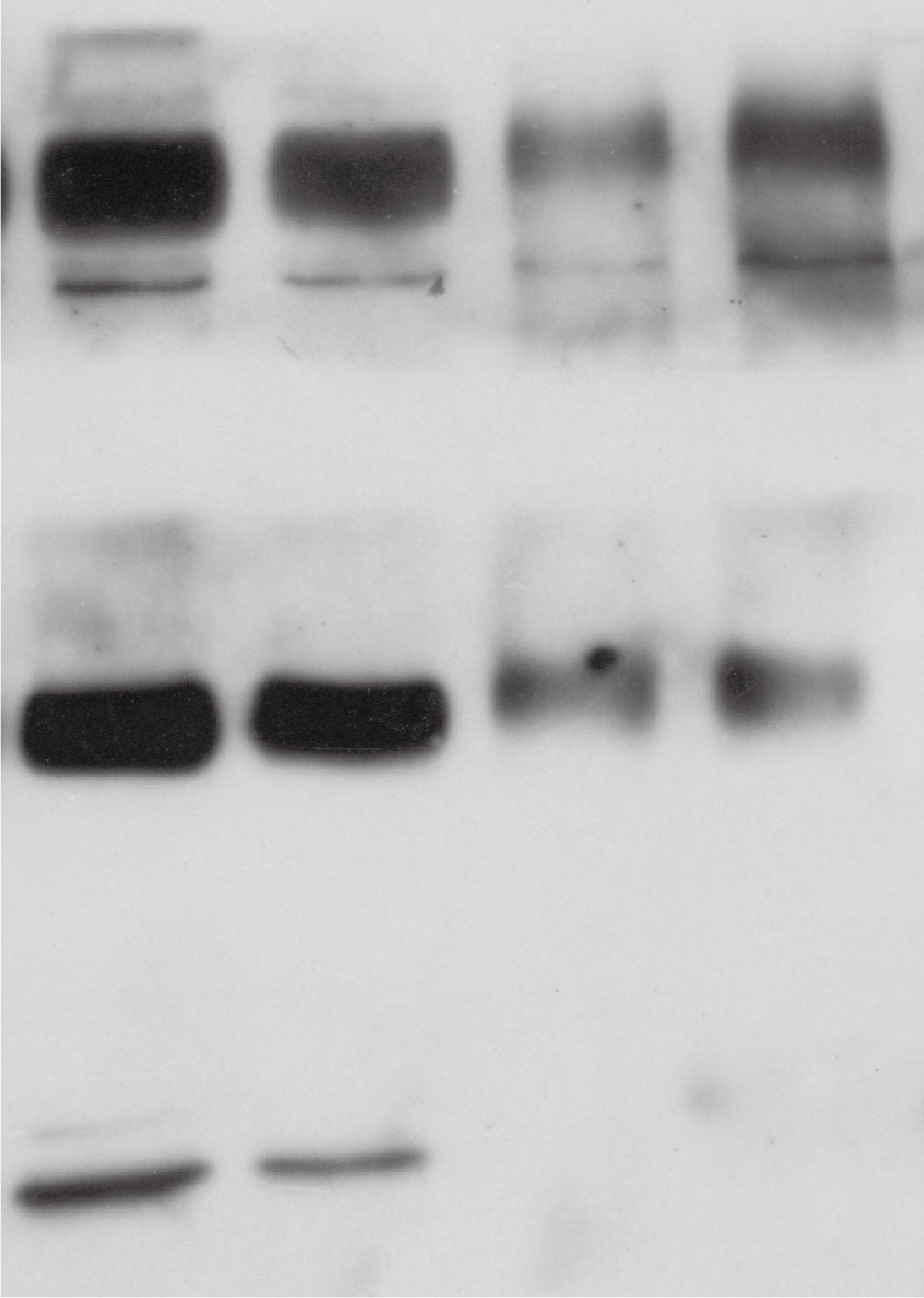

Supplement: Figure 2—source data 2. [file elife-89613-fig2-data2.zip › WB from fig2d.png]

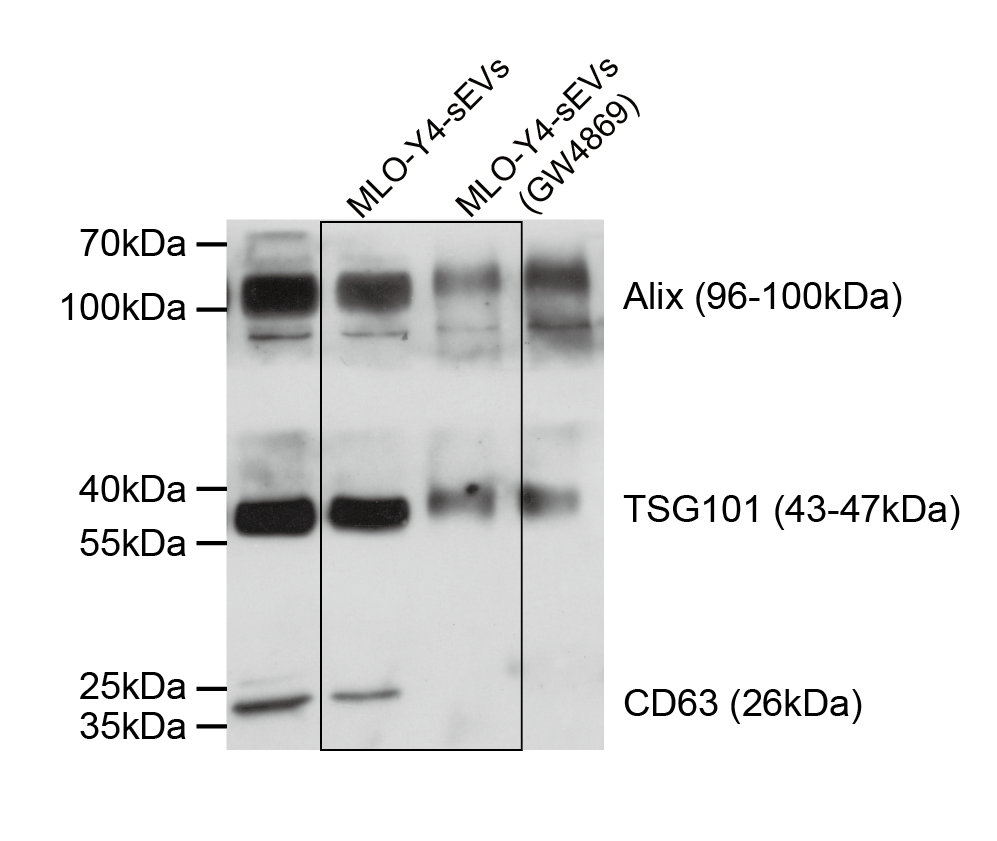

Supplement: Figure 2—source data 3. [file elife-89613-fig2-data3.zip › Figure 2d.png]

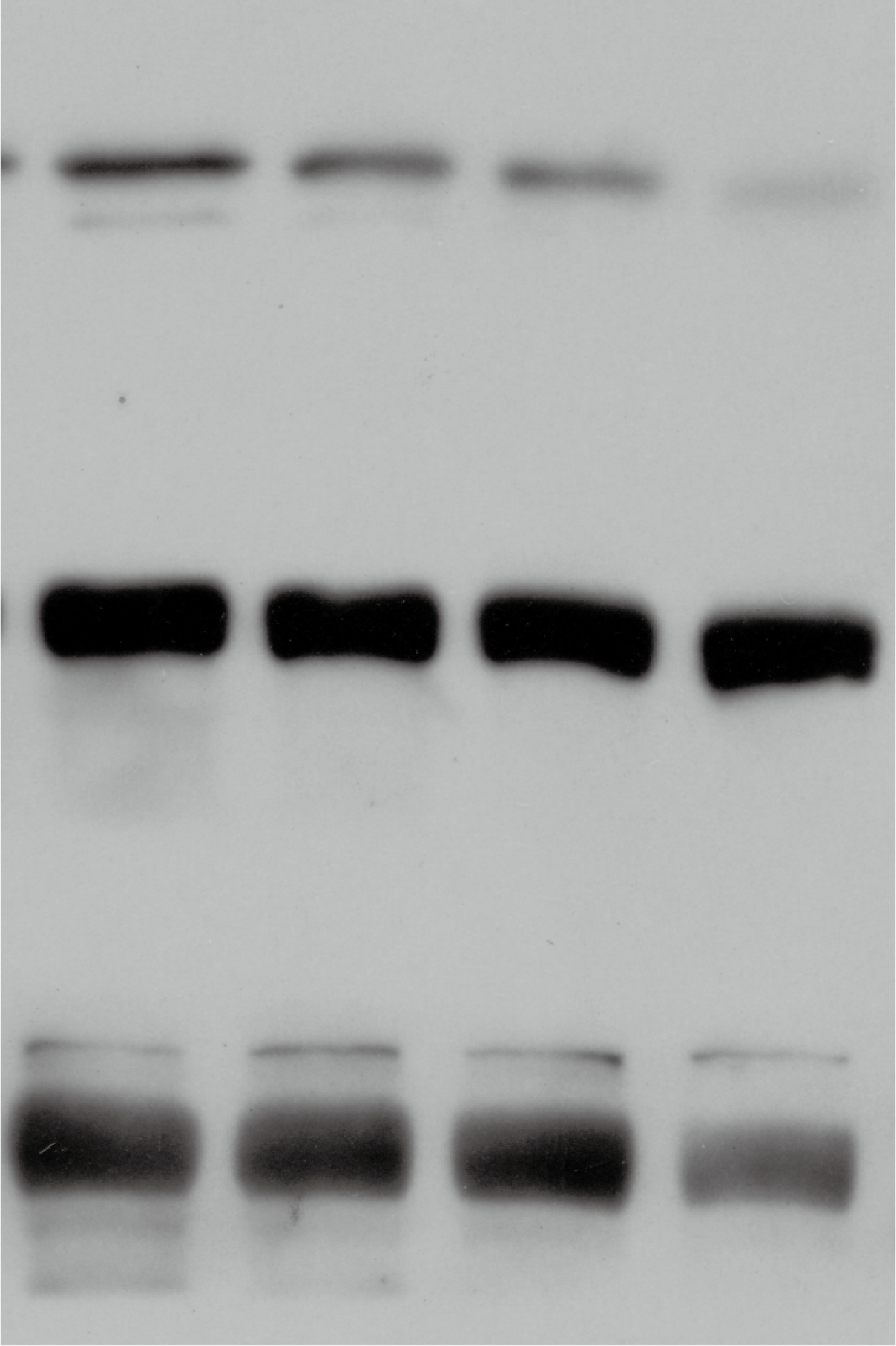

Supplement: Figure 2—figure supplement 1—source data 2. [file elife-89613-fig2-figsupp1-data2.zip › WB from figS2c.png]

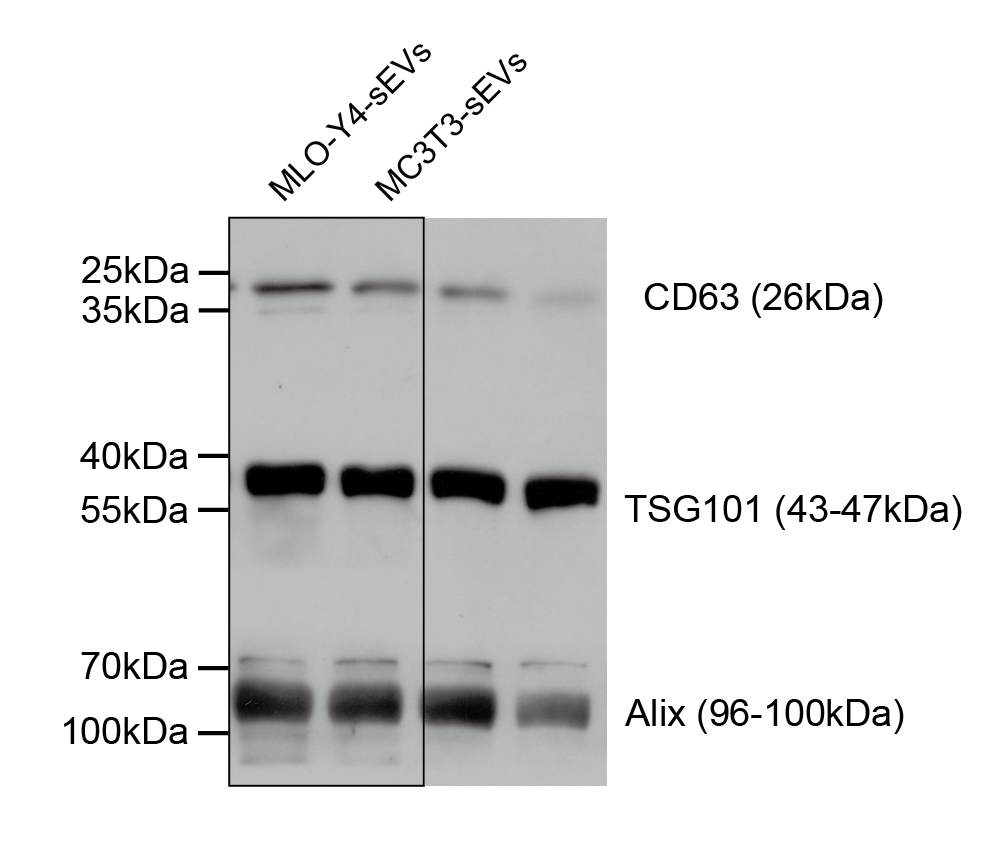

Supplement: Figure 2—figure supplement 1—source data 3. [file elife-89613-fig2-figsupp1-data3.zip › Figure S2c.png]

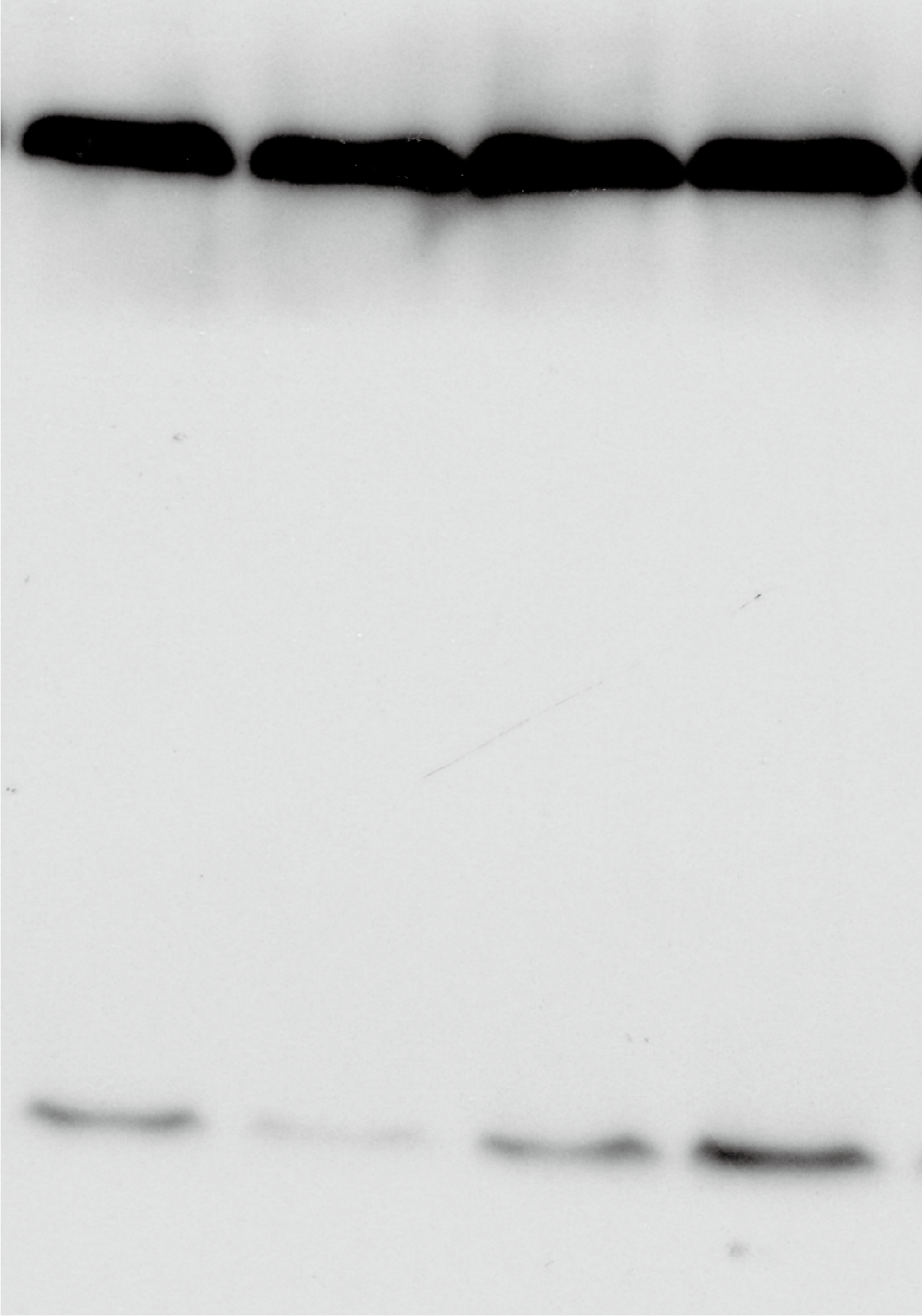

Supplement: Figure 3—source data 2. [file elife-89613-fig3-data2.zip › WB from fig3f.png]

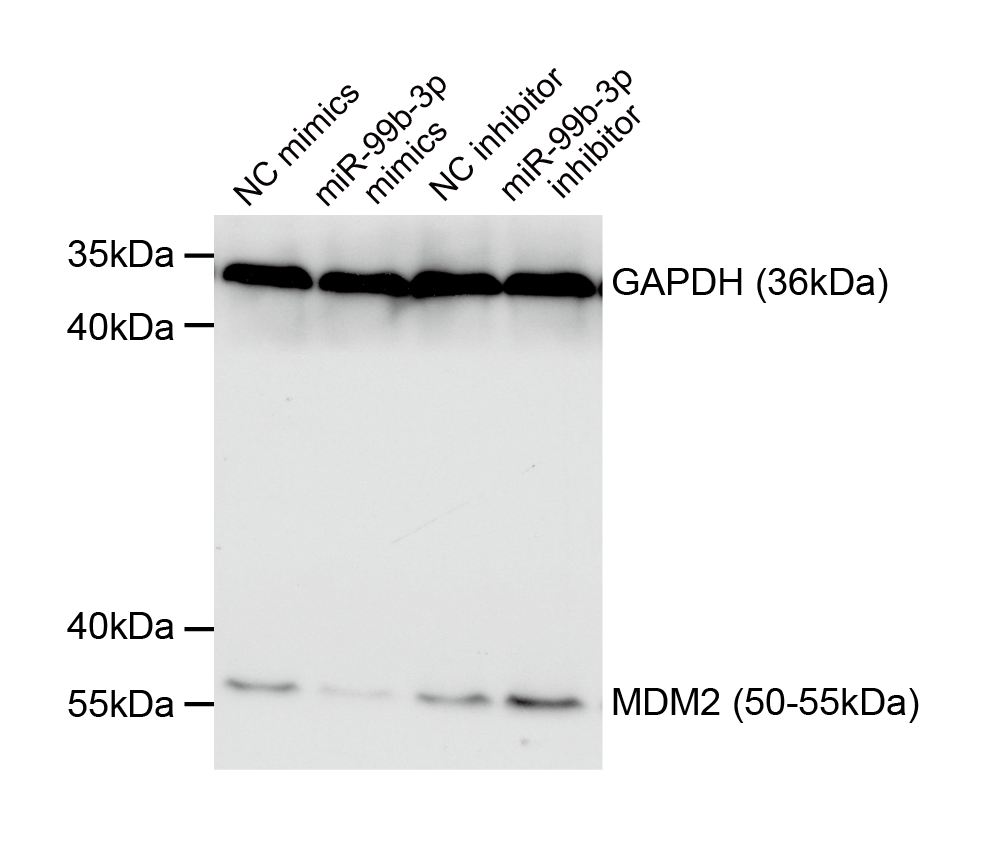

Supplement: Figure 3—source data 3. [file elife-89613-fig3-data3.zip › Figure 3f.png]

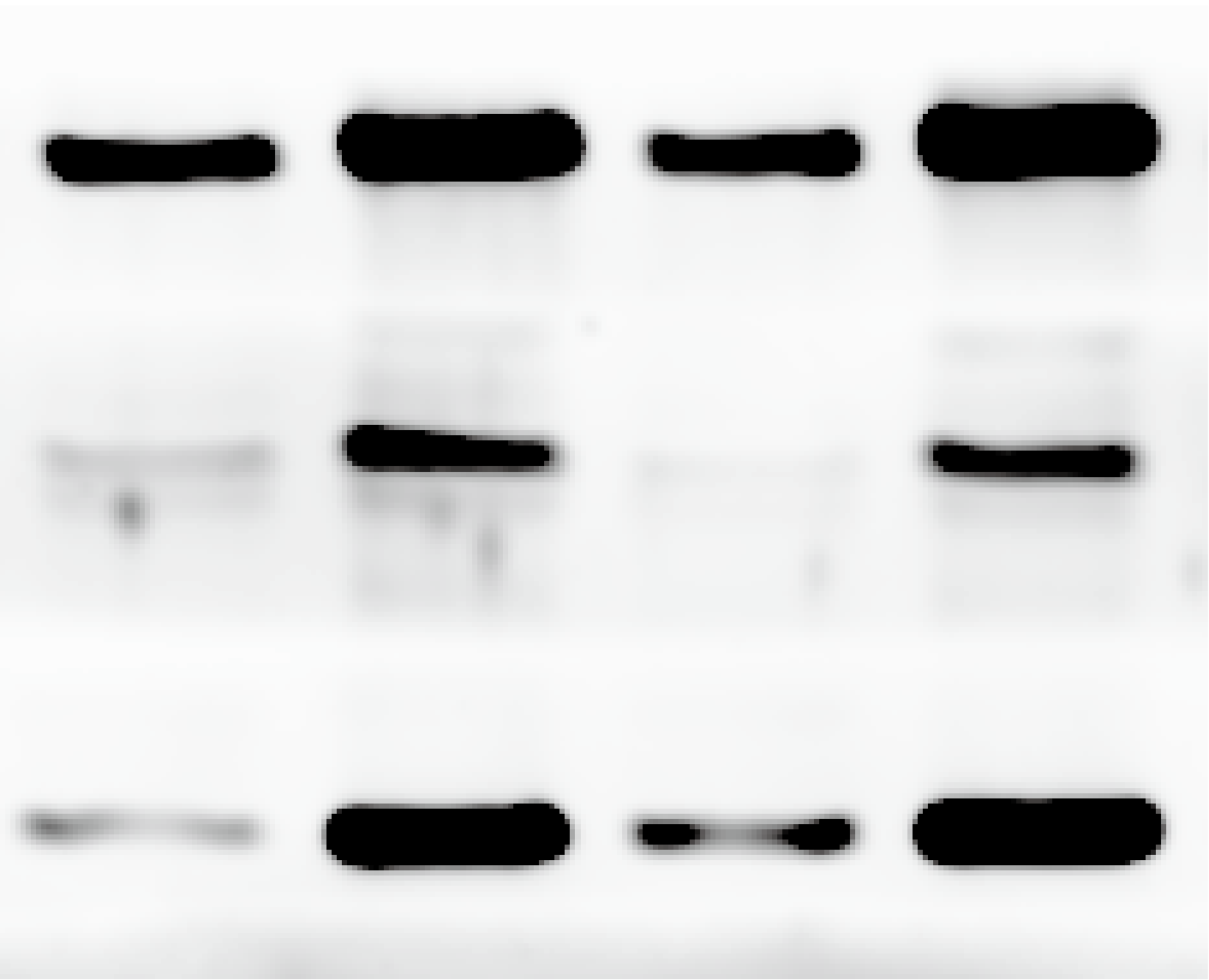

Supplement: Figure 4—source data 2. [file elife-89613-fig4-data2.zip › WB from fig4c.png]

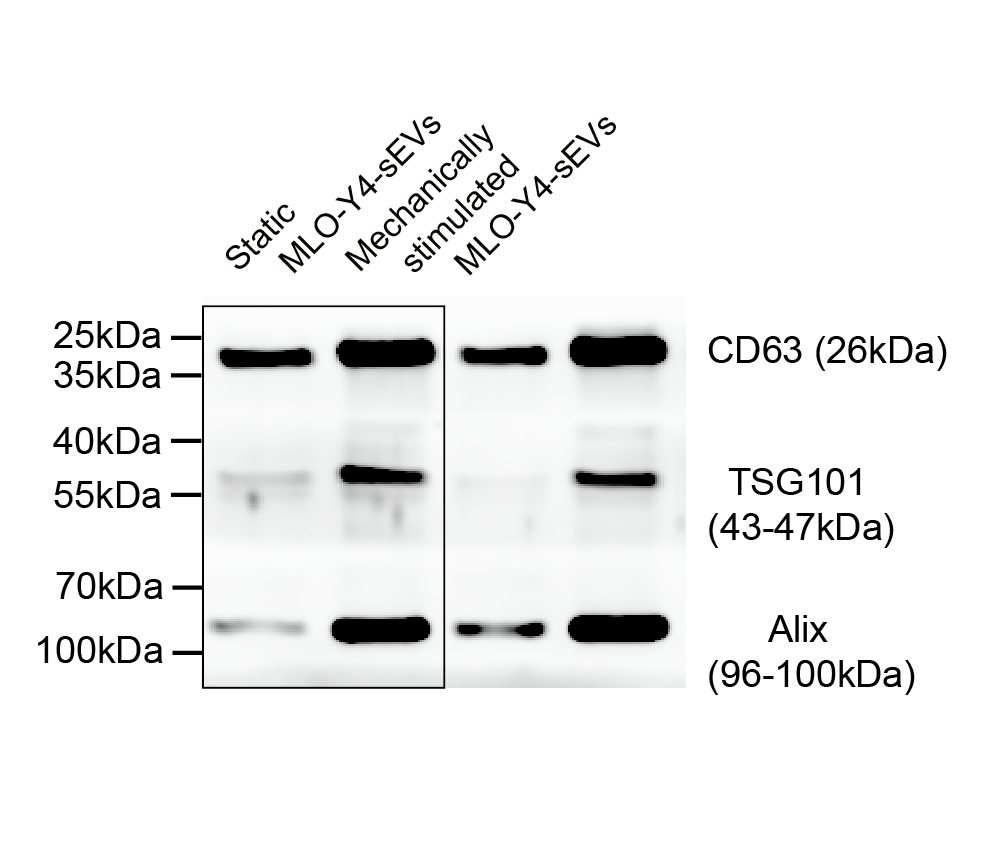

Supplement: Figure 4—source data 3. [file elife-89613-fig4-data3.zip › Figure 4c.png]
